# Sustainable care quality improvement: a scoping literature review of performance measurement in lean healthcare implementations

**DOI:** 10.1186/s12913-025-13598-5

**Published:** 2025-11-07

**Authors:** Caterina Pozzan, Anna Tiso, Chiara Pamich, Chiara Verbano

**Affiliations:** Department of Management and Engineering, University of Padova, Stradella San Nicola 3, 36100 Vicenza, Italy

## Abstract

**Background:**

The phenomenon of demographic changes and the aging population requires Territorial Ambulatory Healthcare (TAH) to provide increasingly high-quality and sustainable care to a growing number of individuals with chronic diseases. In this context, Healthcare Lean Management (HLM) can support sustainable care quality, increasing patient satisfaction and process performance. According to the literature, a significant barrier to sustainability is the limitation of monitoring systems for process performance. For this reason, this study aims to provide a comprehensive overview of the most pursued improvement objectives, performance measurement indicators, and data collection tools and techniques of HLM implementations within TAH.

**Methods:**

Following the PRISMA protocol, a scoping literature review was conducted to investigate the linkages among improvement objectives, performance measurement indicators, and data collection tools and techniques.

**Results:**

The current study provides valuable insights into the performance measurement indicators that should be adopted according to the specific improvement objectives. It highlights that measuring and monitoring are essential activities but often overlooked in HLM projects, negatively affecting the effectiveness of this managerial approach in terms of sustainability over time.

**Conclusions:**

The final results demonstrate how research about performance measurement systems in HLM projects is still in its early stages, both from a theoretical and empirical perspective. The current research enriches the knowledge about the theme and provides managerial support to the journey of territorial care services toward sustainable care quality.

**Supplementary Information:**

The online version contains supplementary material available at 10.1186/s12913-025-13598-5.

## Introduction

The phenomenon of an increasingly aging population is spreading worldwide, and despite demographic disparities, it reflects advances in social and economic conditions. According to the World Health Organization, & United Nations Children’s Fund [1], the global number of people aged 60 years or older is expected to increase by 50% in the coming decades, rising from 1 billion in 2019 to 2.1 billion by 2050. This demographic change requires government authorities and healthcare organizations to manage a growing number of individuals with chronic diseases. To effectively address this challenge, continuous and integrated Long-Term Care (LTC) should be promoted [2]. LTC encompasses all actions designed *“to ensure that people with or at risk of a significant ongoing loss of intrinsic capacity can maintain a level of functional ability consistent with their basic rights, fundamental freedoms and human dignity”* [3] and identifies a complex care system that involves not only health workers but also formal and informal caregivers and social workers [4]. Therefore, an integrated multidisciplinary network that offers accessible care is essential to foster a healthy aging population. Among the primary objectives is the promotion of integrated patient-centered healthcare assistance at the community level within the territorial network [1, 5, 6]. In this context, the role of Territorial Ambulatory Healthcare (TAH) is particularly relevant for managing LTC pathways and the complex needs of the elderly population. Providing adequate care within the outpatient setting can effectively prevent the exacerbation of many chronic diseases, thereby delaying the deterioration of patient conditions and reducing the need for hospitalizations. Furthermore, the territorial network approach facilitates proximity care, enabling healthcare workers to address the individual needs of patients [7]. Conventionally, outpatient services are classified as those that do not require hospitalization (unlike inpatient services), with a clear distinction between different intensity levels of care. Specialized secondary care is primarily provided in outpatient hospital departments rather than in territorial settings, which is typical for primary ambulatory healthcare [8]. To avoid overburdening hospital facilities and support LTC pathways and chronic patients, an emerging TAH model combines primary ambulatory care with low-complexity secondary ambulatory care. This approach facilitates local services to patients who do not require sophisticated hospital infrastructure and equipment or are not in acute emergency conditions [9, 10].

Given the importance of TAH, healthcare organizations must ensure high-quality care within this setting. According to the World Health Organization (2), the quality of care encompasses a broad definition, including efficient and timely services, effective clinical outcomes, integrated and patient-centered assistance, accessibility, equity, and the provision of safe care. Despite the urgency of providing high-quality care, achieving this goal is challenging and requires the implementation of structured approaches that involve leadership and health workers at all levels. These approaches should facilitate the development of learning systems to support the sharing of best practices and sustain improvement over time. Furthermore, ensuring high standards of care requires the adoption of effective measurement systems that support the organization in pursuing its quality objectives [11]. A widely adopted managerial approach to improve process performance and care quality is Healthcare Lean Management (HLM), which aims to enhance patient value by reducing waste, thereby improving care pathways and patient satisfaction. [12, 13, 14]. However, despite the urgency and relevance of implementing HLM in this context, the literature highlights the territorial context as an emerging and innovative setting of application that needs further research to be explored [15, 16]. In particular, the sustainability of these initiatives is a critical issue, as it involves not only successfully completing the implementation but also ensuring continuous monitoring of the improvements achieved over time [17, 18]. As a core principle of HLM, determining success and improvement, as well as supporting ongoing monitoring, requires the use of structured data collection methods and performance measurement indicators [19]. The current literature provides several pre- and post-implementation measurements, but few cases report long-term monitoring results, especially considering the territorial context [16, 20].

For these reasons, the primary purpose of this study is to assess the current state of the art regarding the adoption of specific practices to guarantee implementation sustainability, supporting clinical leadership and managers in expanding the adoption of HLM beyond hospital boundaries and enhancing the role of territorial care. Considering TAH as an innovative context for HLM implementations, the target setting has been expanded due to the limited number of theoretical and empirical studies on this topic. It was extended to include ambulatory services with similar characteristics. Consequently, ambulatory services provided within hospital departments were also included if they could be classified as low- complexity. Therefore, the defined target encompasses all primary and secondary low-complexity outpatient healthcare (LCOH) services. In this study, secondary low-complexity outpatient care refers to any ambulatory service that can be delivered in a territorial setting or an outpatient hospital department without requiring inpatient facilities, emergency services, or surgical activities. These services include diagnostic procedures and clinical activities such as specialist consultation, treatments, and rehabilitation services. Primary care ambulatory services have been included, encompassing visits to general practitioners and pediatricians, as well as health prevention and promotion activities [1, 21, 22].

Therefore, this study aims to provide a comprehensive overview of the improvement objectives, performance measurement indicators, and data collection tools and techniques adopted in HLM implementations within LCOH.

The paper is organized into five additional sections. Section “Sustainable healthcare Lean management implementation in territorial ambulatory healthcare” presents an overview of sustainable HLM implementation in TAH, detailing the motivations that extend beyond the research aim. Section “Methods” presents the research questions and describes the methodology adopted to conduct the scoping literature review. Section “Results” presents the results of the descriptive and content analysis of the database, while Section “Discussion” presents the discussion of the results. Finally, the conclusions are summarized in Section “Conclusion”.

## Sustainable healthcare lean management implementation in territorial ambulatory healthcare

Originating in the automotive industry in the mid-20th century, Lean Management quickly spread to other manufacturing sectors with the purpose of eliminating waste to increase process performance and meet customer needs. Since the early 2000s, it has been applied in the healthcare sector, initially in the United States and then at the international level [19, 23, 24]. HLM is a managerial approach that aims to improve patient value by reducing waste to benefit care pathways and patient satisfaction. To address this purpose, a cultural change and specific tools and techniques are needed to foster continuous improvement [14, 25, 26]. Over the years, this approach has broadened its application beyond a few standardized processes to include several hospital departments and, less frequently, the entire healthcare organization. According to the literature, the territorial context is an innovative setting of application that needs further research to be explored [15, 16]. In addition, its implementation has, in some cases, integrated other managerial approaches to meet the growing demand for high performance and quality of care. Notable examples include its integration with Six Sigma methodologies and Clinical Risk Management (CRM) [14, 27]. In particular, the integration of CRM and HLM can support health organizations in proactively reducing waste and risks, thus preventing adverse events for patients, which is critical to ensuring high levels of care quality [11, 28–30].

The HLM improvement approach focuses on implementing small, incremental changes that, over time, drive a profound and comprehensive transformation within the organization, particularly in terms of cultural aspects. Through iterative cycles, cultural, managerial, and organizational improvements are introduced, consolidated, and standardized into the routine activities of an organization. This philosophy of continuous improvement supports the sustainability of improvements over time by engaging the entire organization and fostering a solid personal commitment among employees [26]. In this context, sustainability refers to the preservation and maintenance of performance improvements and cultural changes over time. According to Henrique et al. [31], lean sustainability comprehends three key aspects: lean tools (i.e., work standardization, A3 method, key performance indicators, kaizen events, visual management boards, gemba walk), lean methods (i.e., PDCA, DMAIC) and lean people (i.e., employee involvement, team training). However, achieving HLM sustainability within the healthcare sector remains challenging due to cultural, organizational, and managerial aspects that characterize this context. According to the literature, one of the most significant barriers is the limited monitoring systems that support ongoing key performance indicator measures through effective data collection, storage, and analysis. Indeed, despite the recognized success of HLM projects in enhancing efficiency and timeliness, ensuring the long-term sustainability of their improvement outcomes represents one of the main limitations of this approach. To promote sustainable continuous improvement, critical aspects of HLM include adopting a process-oriented approach, the availability of measurement indicators, access to data, setting clear and specific project goals, employing structured problem-solving approaches such as DMAIC, and implementing performance measurement systems to ensure effective measurement and monitoring of results over time [31]. Thus, improvement cannot be confirmed unless it can be measured [19]. The obstacles in accessing process data frequently arise from incomplete information recording, inadequate data collection protocols, obsolete or non-integrated information systems, and lack of personnel training. It is crucial to carefully plan data collection, support a performance measurement system to monitor target improvement objectives, and design an approach tailored to the specific requirements [32–34]. Few previous studies focused on these aspects, leaving room for further investigation [35]. These considerations become even more significant when applied to a complex and relatively unexplored context such as TAH.

## Methods

Considering the gaps identified in the literature and the objectives of this study, the following research questions were framed:

### RQ1:

What are the care quality improvement objectives of HLM implementations within the LCOH setting?

### RQ2:

What are the performance measurement indicators and data collection tools and techniques adopted during HLM implementations within the LCOH setting?

**Fig. 1:**
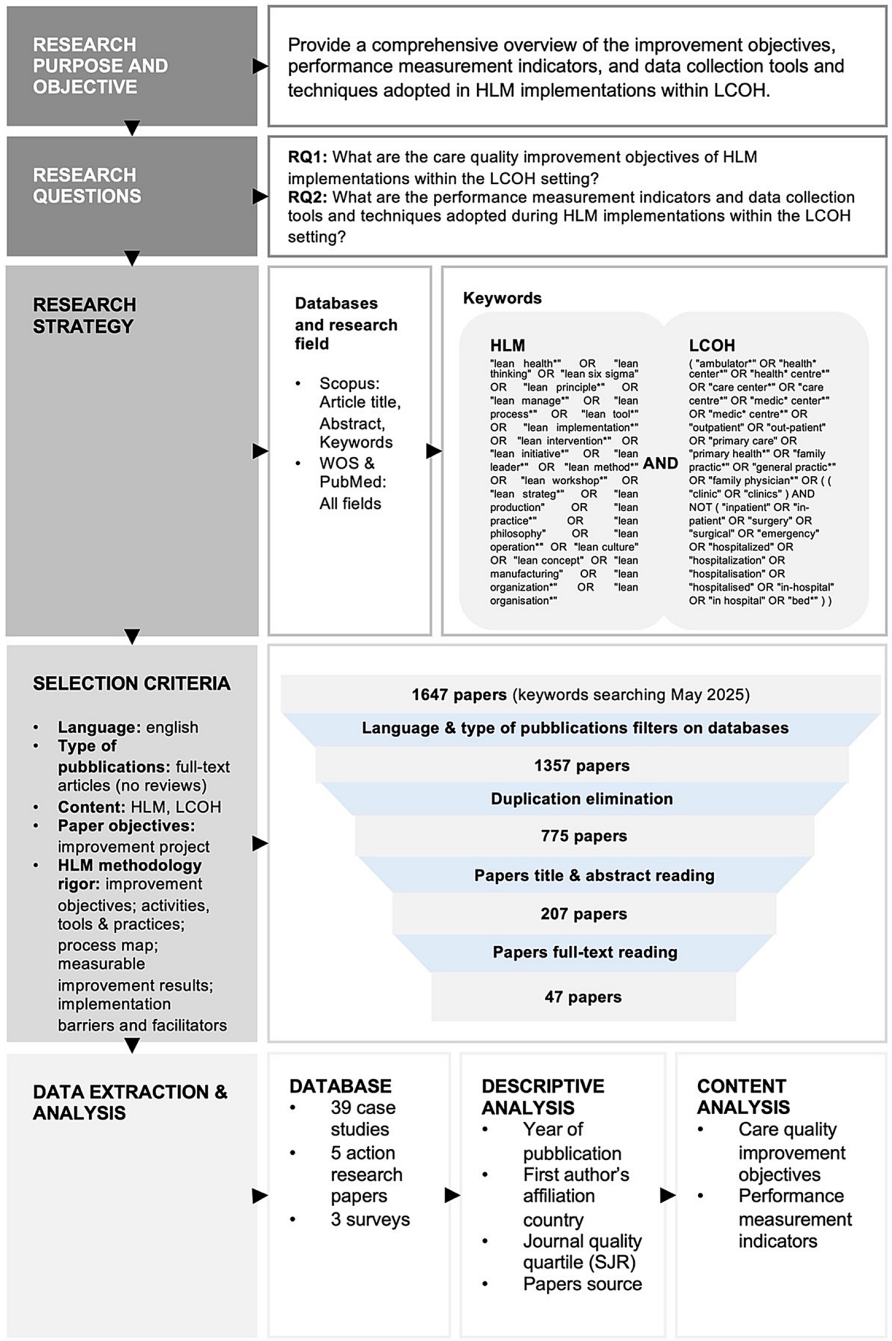
Research protocol

A scoping literature review was conducted to address these research questions, following the PRISMA protocol [36], as illustrated in Fig. 1. Three academic databases (Scopus, Web of Science, and PubMed) have been consulted. The search activity has been performed in the ‘Article title, Abstract, Keywords’ field for Scopus and ‘All fields’ for Web of Science (WOS) and PubMed. The search query was defined by combining HLM and LCOH keywords, as shown in Fig. 1. The search was conducted in May 2025 and yielded 775 papers after applying language and document type filters within the databases and eliminating duplicates. Only full-text articles in English were included, while reviews were excluded.

Two authors independently conducted the following screening of titles, abstracts, and full-text articles; any divergences were discussed with the other two authors and resolved. The entire searching and screening process was jointly reviewed and verified by all four authors to ensure methodological rigor and consistency.

In details, title and abstract screening excluded 568 papers, and the subsequent full-text reading eliminated 160 papers according to content inclusion criteria. Considering both the screening of titles and abstracts as well as full-text review, 450 papers were excluded for not considering LCOH as a target setting. This included studies that did not explicitly focus on outpatient care, as well as those involving hospitalized patients or complex care services that require hospital-based infrastructure, even when provided on an outpatient basis, such as day hospital surgery or emergency care services. An additional 156 papers were excluded as they did not address HLM. This included studies that claimed to use HLM but failed to report any improvement activities or specific tools and techniques. Moreover, 60 papers were excluded due to insufficient evidence of a rigorous HLM implementation methodology. Specifically, these studies failed to report at least two of the following key elements: clearly stated improvement objectives; improvement activities, tools, and practices; process mapping; measurable improvement results; and the identification of barriers and facilitators to healthcare quality improvement. Finally, 39 studies were removed as they were not available as full-text academic articles, 22 were excluded because they did not aim to improve healthcare quality, and 1 paper was excluded because it was not written in English. This resulted in a final database of 47 papers, comprising 39 case studies, 5 action research papers, and 3 surveys (see Additional file 1).

The database was analyzed by performing both descriptive and content analysis. The descriptive analysis encompasses the year of publication, the quality of the articles based on journal quartile classification, and the countries of affiliation of the authors to highlight the most involved nationalities. The content analysis focused on the care quality improvement objectives, performance measurement indicators, and data collection tools and techniques adopted to measure the achieved improvements.

## Results

**Fig. 2:**
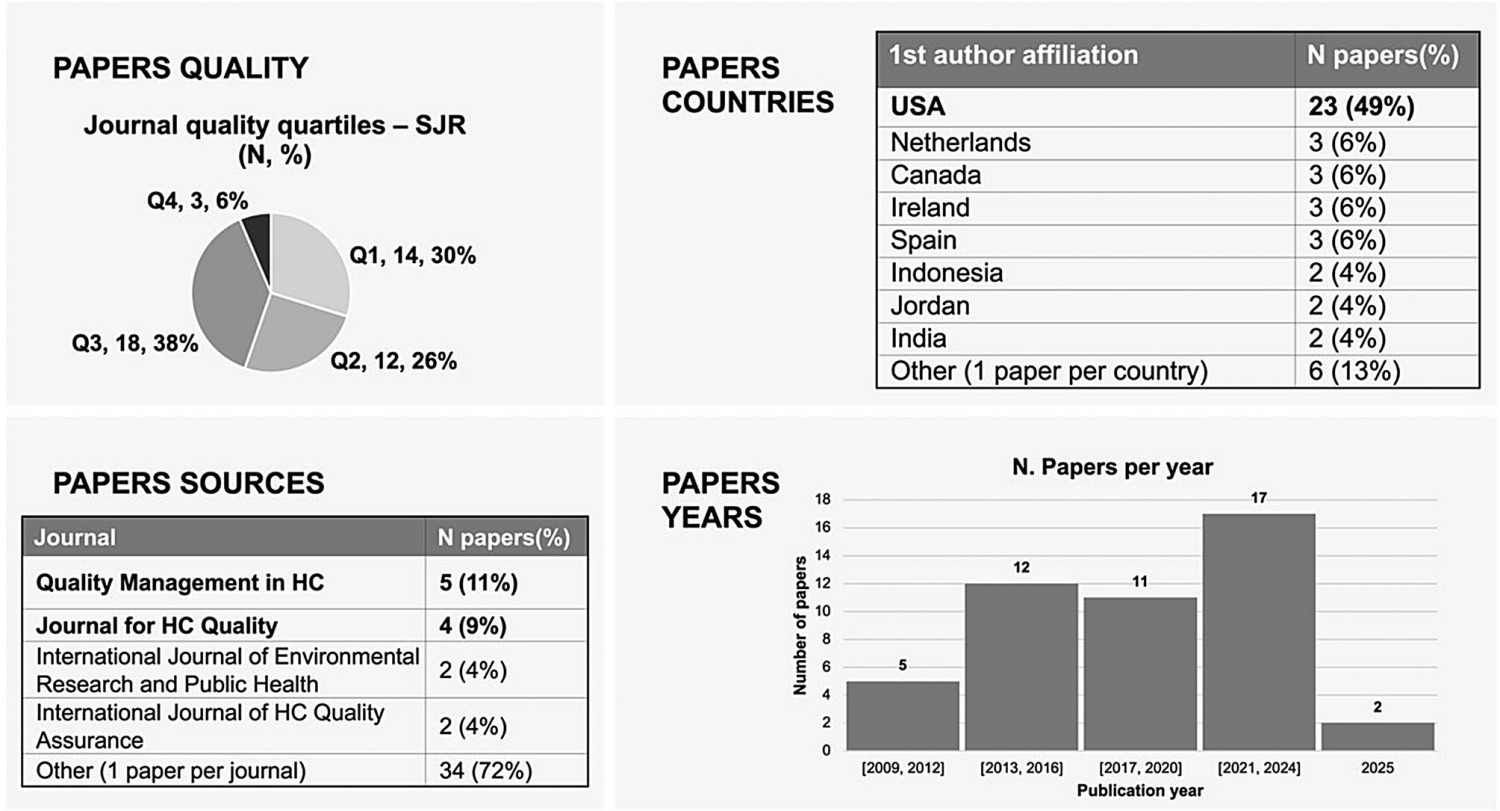
Descriptive analysis of the literature

A descriptive analysis was conducted on all the included articles to provide an overview of the quality of selected articles, the publication year, and the most active countries in publishing empirical papers on HLM implementations within the LCOH setting. The Scimago Journal & Country Rank (SJR) was adopted to evaluate the quality of the journals. SJR is *“a publicly available portal that includes the journals and country scientific indicators developed from the information contained in the Scopus database”* [37]. As shown in Fig. 2, more than half of the articles are published in journals classified as Q1 or Q2 in terms of quality quartiles, and 94% of the articles belong to the Q1, Q2, and Q3 quartiles, indicating a high quality of the extracted database. Additionally, excluding articles that did not present rigorous evidence on the HLM implementation methodology ensured high-quality literature for research purposes. Analysis of the publication years shows a positive upward trend from 2009 to 2025. Furthermore, the actual number of papers published between 2021 and 2025 is expected to be higher than reported, as not all 2025 publications were available in Scopus, WOS, and PubMed at the time of the keywords search. This highlights a growing interest in the research topic. The first author’s country of affiliation analysis reveals that the United States is strongly dominant, accounting for 49% of the selected articles. This suggests limited empirical evidence exists on HLM implementation in LCOH settings outside the United States, such as in European public healthcare organizations.

The content analysis was conducted on all the included articles. It investigates the care quality improvement objectives, performance measurement indicators, and data collection tools and techniques adopted during HLM improvement projects within LCOH. Performance measurement indicators are classified according to care quality categories, highlighting their relationship with care quality improvement objectives and data collection tools and techniques used to assess the improvements achieved. In the following, the results are presented according to the research questions.

### What are the care quality improvement objectives of HLM implementations within the LCOH setting? (RQ1)

Table 1 and Table 2 present care quality improvement objectives, categorized according to relevant care quality outcomes: timeliness, efficiency, patient-centeredness, effectiveness, employees’ work-balance, and safety.

**Table 1:** Timeliness and efficiency quality improvement objectives and performance measurement indicators

Care quality improvement objectives	Performance measurement indicators	Definition/description	References
**TIMELINESS** • Decreasing Lead Time • Decreasing Waiting Time • Providing timely care	Lead time (20 papers)	Total time required for patients to complete a single ambulatory visit	[38–57]
	Waiting time (18 papers)	Patients waiting times between two different tasks (clinical or administrative) during an ambulatory visit	[38–42, 46, 47, 50, 52, 53, 55, 56, 58, 59, 60–63]
	Cycle time (16 papers)	Total time required for patients to complete a single task (clinical or administrative) during an ambulatory visit	[38, 40–42, 44, 46, 47, 49, 50, 53, 55–57, 60, 63, 64]
	Time to care (6 papers)	Patients waiting time between appointment request or booking and the actual visit	[65–70]
	Waiting queue length (4 papers)	Number of patients waiting for care assistance within the facility that provides the ambulatory visit	[50, 59, 64, 65]
	Total throughput time (3 papers)	Total time required for patients to complete a care pathway, which can involve multiple ambulatory visits distributed over time	[69, 71, 72]
	N. Patient tasks (2 papers)	Number of different tasks (both clinical and administrative) experienced by patients during an ambulatory visit	[40, 71]
**EFFICIENCY** • Improving process standardization • Increasing productivity • Reducing intake appointments no-shows • Sustaining workflow redesign over time	N. Patients (6 papers)	Number of patients treated calculated over a certain period, either in absolute terms or divided by the number of care providers or workstations	[45, 47, 53, 54, 69, 73]
	Value-added ratio – VAR (5 papers)	Total time required for patients to complete value-added tasks (value-added time*) divided by lead time. *Value-added time: total time to complete all tasks required to add value from the patient’s perspective	[38, 40, 50, 56, 74]
	N. Scheduled appointments (4 papers)	Number of scheduled visits or phone consultations per period	[67, 69, 71, 75]
	% Task occurrence (4 papers)	Percentage of occurrences of a particular task out of the total number of appointments or patients over a certain period	[63, 75, 76, 77]
	N. Nurses (2 papers)	Number of nurses calculated over a certain period either in absolute terms or divided by the number of patients treated	[45, 73]
	N. or % No-shows (2 papers)	Number or percentage of appointments in which patients did not attend out of the total number of appointments scheduled over a certain period	[66, 71]
	N. Unscheduled appointments (2 papers)	Number of unscheduled visits or telephone consultations per period	[67, 75]
	Overall Resource Efficiency – ORE (1 paper)	Number of patients treated per period divided by potential capacity** **Potential capacity: physicians’ total time available divided by visit time per patient	[69]
	N. Patient tasks (1 paper)	Number of different tasks (both clinical and administrative) experienced by patients during an ambulatory visit	[45]
	N. Recurrent appointments (1 paper)	Number of repeated visits due to procedural errors per period	[68]
	% Compliance with new guidelines (1 paper)	Percentage of time the new guidelines were adhered to by care providers out of the total time required to complete care tasks	[55]
	Perceptive process efficiency index (2 papers)	Survey administered to employees to assess productivity and workflow redesign sustainability	[78, 79]

***A single paper may reference multiple care quality improvement objectives and performance measurement indicators

**Table 2:** Patient-centeredness, effectiveness, employees’ work-balance, and safety quality improvement objectives and performance measurement indicators

Care quality improvement objectives	Performance measurement indicators	Definition/description	References
**PATIENT-CENTEREDNESS** • Improving patients’ satisfaction	Perceptive patients’ satisfaction index (7 papers)	Patients’ satisfaction survey administered to patients or employees	[39, 45, 55, 59, 61, 78, 80]
**EFFECTIVENESS** • Enhancing diseases prevention • Increasing disease diagnoses	N. or % Prevention activities (4 papers)	Number or percentage of visits during which prevention activities (screening, vaccination, follow-up, etc.) are performed out of the total number of visits over a certain period	[57, 81–83]
	N. New diagnoses (1 paper)	Number of new diagnoses for a specific condition over a certain period	[84]
**EMPLOYEES’ WORK-BALANCE** • Improving employees’ work experience satisfaction	% Absenteeism (1 paper)	Percentage of employees’ actual working time out of the total expected working time over a certain period	[73]
	N. Overtime (1 paper)	Number of overtime hours worked by employees within a certain period	[73]

**Fig. 3:**
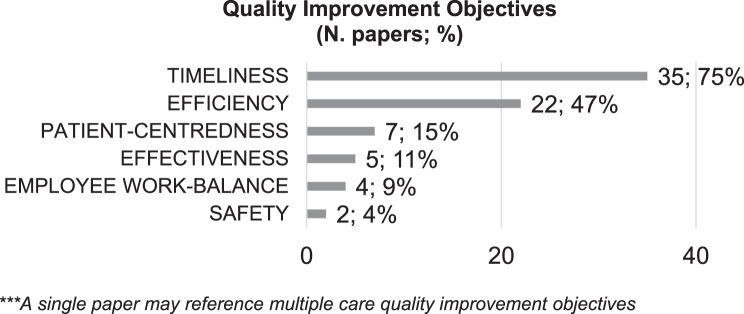
Quality improvement objectives

Observing the improvement objectives assessed through performance measurement indicators and highlighted in Fig. 3, it can be concluded that most of the empirical evidence (35 papers) focuses on enhancing timeliness by decreasing Lead Time, Waiting Time and providing timely care. The efficiency category is also well-represented, with 22 papers reporting projects aimed at enhancing process standardization, increasing productivity, and reducing the no-shows rate for intake appointments. Notably, this category includes a project that aims to sustain workflow redesign over time, standing out as the only example in the reviewed literature explicitly targeting the long-term sustainability of improvements [78]. Regarding the other care quality categories, there is limited evidence on patient-centeredness, effectiveness, employees’ work balance, and safety. Among these, significant attention is given to patient experience, with seven papers addressing patient satisfaction and two papers focusing on patient safety.

### What are the performance measurement indicators and data collection tools and techniques adopted during HLM implementations within the LCOH setting? (RQ2)

**Fig. 4:**
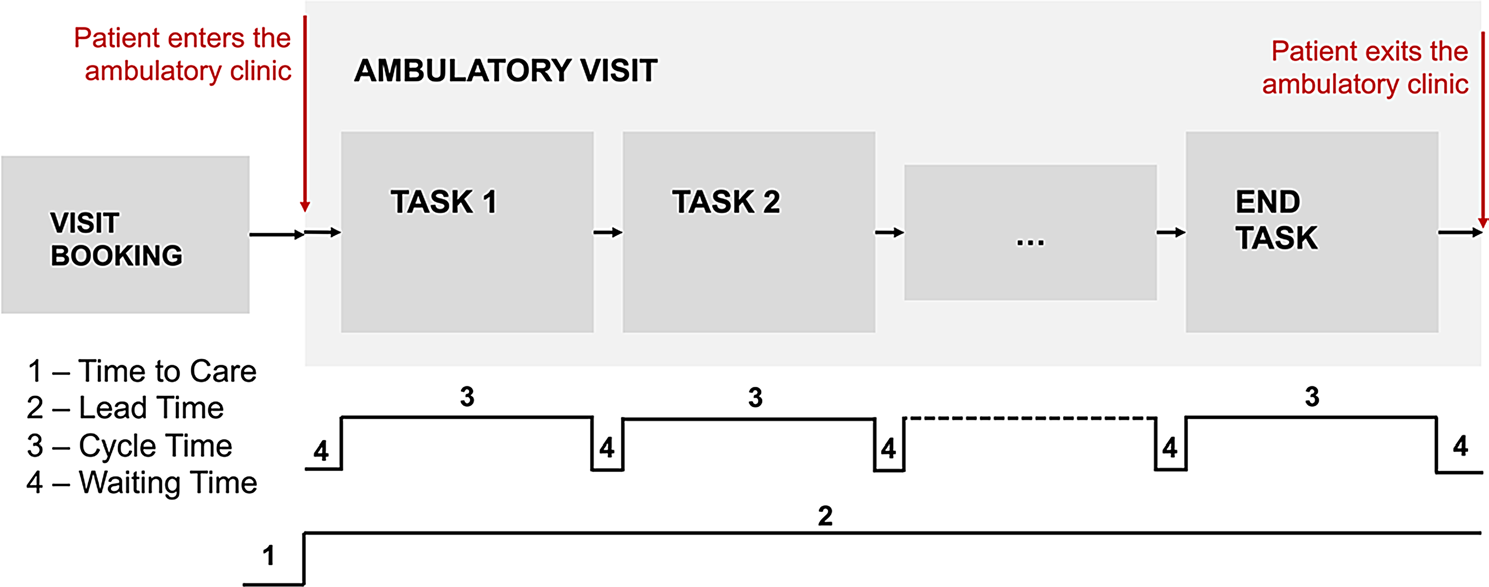
Ambulatory visit time indicators definition

Tables 1 and 2 offer a detailed explanation of each performance measurement indicator in terms of definitions and references from the selected articles. Unique labels were identified to properly organize the various metrics, recognizing that, despite measuring similar aspects, they can be named differently in the literature. This is particularly evident when considering timeliness indicators. Figure 4 visually represents the time indicators that characterized ambulatory visits. A single ambulatory visit encompasses various tasks performed by both care providers (clinical tasks) and administrative personnel (administrative tasks). Among the studies reviewed, Lead time (20 papers), Waiting time (18 papers), and Cycle time (16 papers) are the most frequently adopted performance measurement indicators for timeliness objectives, each detected in over 30% of the studies. This high frequency is particularly notable, as the following most commonly used indicator (various patient satisfaction indexes measured through surveys administration) is cited in only 7 studies.

Table 3 categorizes indicators based on the improvement objectives to which they are associated, highlighting that not all indicators are adopted uniformly, but rather depend on the target goals. Within the timeliness category, Lead time, Waiting time, and Cycle time are commonly adopted when the objective is to decrease Lead time and Waiting time. Additionally, other indicators such as waiting queue length, the number of tasks experienced by patients, and time to care are also used for this exact purpose. The number of patient tasks, time to care, and total throughput time are further adopted to assess the provision of timely care to patients. Most efficiency performance measurement indicators are associated with objectives aimed at increasing productivity, although they vary significantly, each addressing different aspects. Effectiveness indicators are primarily used to enhance disease prevention and diagnosis, while indicators related to patient-centeredness and employee work-balance are associated with improving patients’ and employees’ satisfaction, respectively. Lastly, concerning safety aspects, two studies measure the number of errors identified within the care pathway to improve patient safety.

**Table 3:** Relationship between performance measurement indicators and care quality improvement objectives

Performance measurement indicators ↓	Care quality improvement objectives ↓			
TIMELINESS	Decreasing Lead Time	Decreasing Waiting Time	Providing timely care	
Lead time (20 papers)	X	X	-	
Waiting time (18 papers)	X	X	-	
Cycle time (16 papers)	X	X	-	
Time to care (6 papers)	-	X	X	
Waiting queue length (4 papers)	-	X	X	
Total throughput time (3 papers)	-	-	X	
N. Patient tasks (2 papers)	-	X	X	
**EFFICIENCY**	**Improving process standardization**	**Increasing productivity**	**Reducing intake appointments no-shows**	**Sustaining workflow redesign over time**
N. Patients (6 papers)	-	X	-	-
Value-added ratio – VAR (5 papers)	-	X	-	-
N. Scheduled appointments (4 papers)	X	X	-	-
% Task occurrence (4 papers)	X	X	-	-
N. Unscheduled appointments (2 papers)	X	X	-	-
N. nurses (2 papers)	-	X	-	-
N. or % No-shows (2 papers)	-	-	X	-
Overall Resource Efficiency – ORE (1 paper)	-	X	-	-
N. Patient tasks (1 paper)	X	-	-	-
N. Recurrent appointments (1 paper)	-	X	-	-
% Compliance with new guidelines (1 paper)	X	-	-	-
Perceptive process efficiency index (2 papers)	-	X	-	X
**EFFECTIVENESS**	**Enhancing diseases prevention**		**Increasing diseases diagnoses**	
N. or % Prevention activities (4 papers)	X		-	
N. New diagnoses (1 paper)	-		X	
**PATIENT CENTEREDNESS**	**Improving patients’ satisfaction**			
Perceptive patients’ satisfaction index (7 papers)	X			
**EMPLOYEES’ WORK-BALANCE**	**Improving employees’ work experience satisfaction**			
% Absenteeism (1 paper)	X			
N. Overtime (1 paper)	X			
Perceptive work experience satisfaction index (3 papers)	X			
**SAFETY**	**Improving patients’ safety**			
N. Errors (2 papers)	X			

***A single paper may reference multiple improvement objectives and performance measurement indicators for each care quality goal

**Fig. 5:**
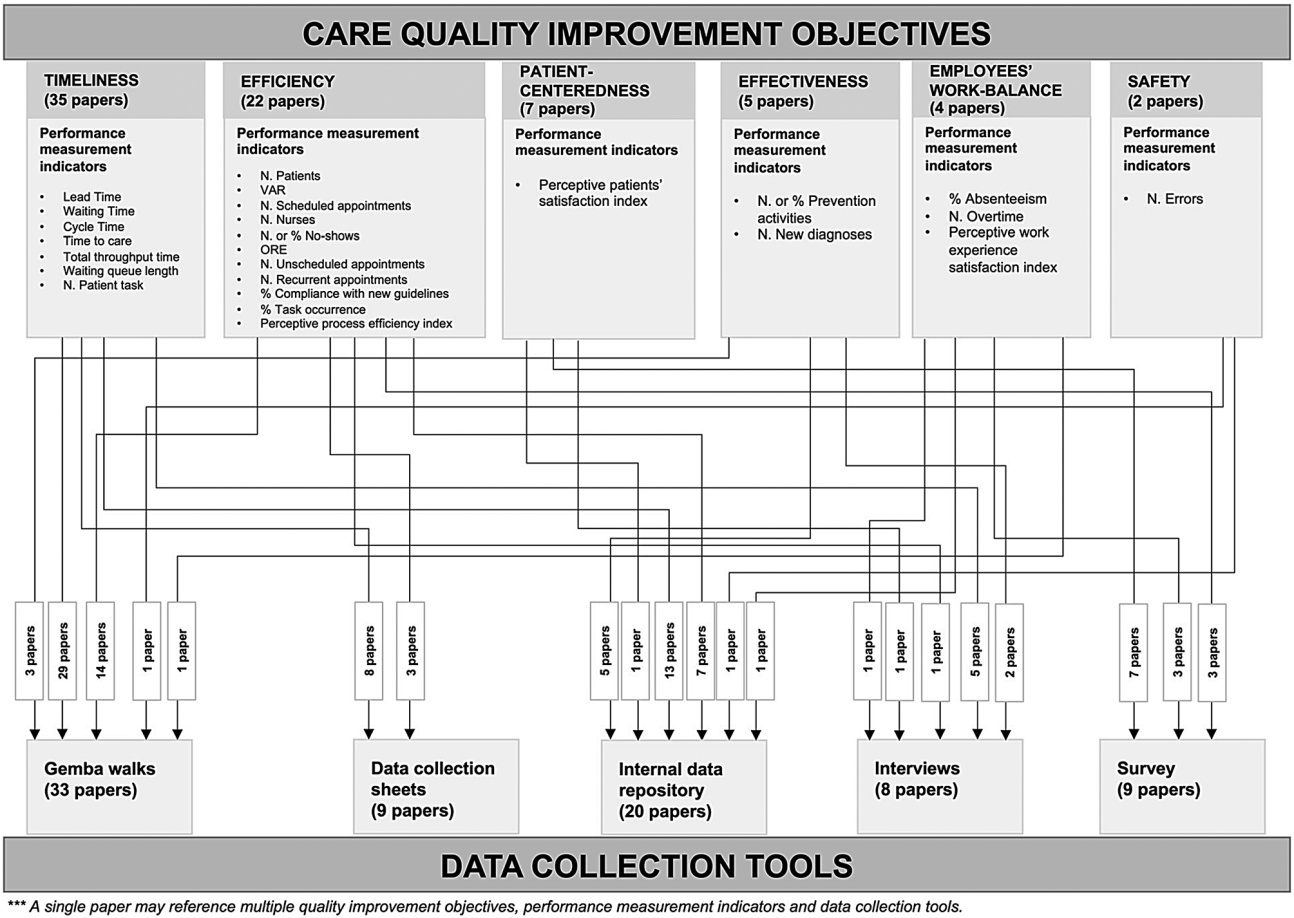
Care quality improvement objectives and data collection tools

The data collection tools and techniques used to measure performance indicators are illustrated in Fig. 5. For the timeliness objectives category, data are primarily collected through field observations (gemba walks), with over 70% of the papers adopting this method. Additionally, 7 papers combine gemba walks with the use of data collection sheets. These tools are also the most widely employed in the efficiency category, with 7 studies utilizing internal data repositories and 3 studies employing surveys for data collection. These results emphasize the scarcity of evidence regarding data collection initiatives that rely on internal data repositories to pursue timeliness and efficiency objectives. Surveys are predominantly used to measure perceptual indexes related to patient and employee satisfaction. Effectiveness is the category in which internal data repositories are most frequently employed: 100% of the papers focused on improving efficiency rely on clinical data from patients’ data warehouses. The two papers addressing safety objectives collect errors through direct observation or retrospectively by analyzing documents and internal database records. Moreover, valuable insights were gained regarding control activities, as illustrated in Table 4. Most of the studies analyzed (79%), conducted monitoring activities within the first 12 months after the implementation of improvement countermeasures. However, fewer than 30% of the papers reported monitoring activities beyond the 12-month mark, and there is minimal evidence of control plans implementing standardized and digitalized procedures for long-term monitoring, such as dashboards that rely on internal data repositories and automatically collected data [72, 80]. Notably, even studies highly relevant to the long-term sustainability of improvements, such as the one by Hung et al. [78], rely on data collection tools like surveys and interviews, which are not easily adaptable to digitalized monitoring processes.

**Table 4:** Improvement monitoring activities

Improvement monitoring period	N papers (%)	Papers
< 12 months	37 (79%)	[39, 41–49, 50, 53–55, 57–59, 61–67, 71, 72, 74–78, 80–84]
≥12 months	13 (28%)	[39, 47, 65–73, 78, 79, 82, 83]

***A single paper may reference to multiple improvement monitoring periods

## Discussion

The following section presents the discussion of the results, addressing the research questions.

### What are the care quality improvement objectives of HLM implementations within the LCOH setting? (RQ1)

Aligning with previous literature, the analysis of care quality improvement objectives highlights the scarcity of empirical evidence on HLM implementations that address care quality objectives beyond efficiency and timeliness. This analysis confirms that integration, patient-centeredness, safety, equitability, effectiveness, and employee-work balance are inadequately addressed, as previously highlighted in earlier studies [16, 20, 85]. Among the underrepresented care quality categories, this study highlights improvement objectives related to employees’ work-balance. Although not explicitly defined by the World Health Organization (2), this category highlights the need to address these issues, especially as many healthcare systems currently face significant human resources constraints, often leading to overburdened and fatigued health workers [86]. Moreover, the limited integration of CRM into HLM implementations is particularly noteworthy. In fact, safety plays a critical role in preventing adverse events and ensuring high-quality care, two urgent aspects to address in primary and territorial care [11, 28, 29, 30].

In light of the improvement objectives discussed, sustainability also emerges as a relevant aspect, as it can encompass a variety of interrelated concepts. When considering the sustainability of HLM implementation, two key dimensions must be considered [17, 18]: successfully completing improvement projects and maintaining these improvements over time. Both are essential to avoid wasting resources and to prevent enhanced processes from reverting to their pre-implementation state. However, despite their critical importance and the urgency of addressing them, there is limited evidence of projects explicitly aiming to achieve sustainable implementation objectives [31, 87]. Beyond the notion of sustainability directly tied to the success of HLM implementation, these initiatives can also contribute to broader sustainability goals, encompassing economic, social, and environmental aspects [88]. While this study does not explicitly focus on cost reduction, it shows that most HLM implementations in LCOH pursue economic sustainability by aiming to improve process timeliness and efficiency through enhanced standardization, increased productivity, and the reduction of non–value-adding activities. Social sustainability also emerges from this analysis. While only partially addressed, it is pursued through objectives such as strengthening prevention and early diagnosis efforts, improving patient safety and satisfaction, and enhancing working conditions for healthcare professionals, specifically by reducing overtime hours and absenteeism rates. Nevertheless, there remains a scarcity of studies employing performance measurement indicators specifically designed to assess clinical outcomes. This result represents a missed opportunity since evaluations are crucial for ensuring the consistent maintenance of care quality standards and enabling meaningful benchmarking practices [89]. Finally, although environmental sustainability has become an increasingly important concern in recent decades, this study found no explicit focus on this aspect. While resource optimization and waste reduction objectives were observed, there were no structured evaluations aimed at defining improvement objectives or performance measurement indicators for assessing environmental impact. This may be because environmental sustainability is still primarily addressed in hospital settings, while its application in the context of HLM implementations in LCOHs remains relatively underexplored [90, 91].

### What are the performance measurement indicators and data collection tools and techniques adopted during HLM implementations within the LCOH setting? (RQ2)

The analysis of performance measurement indicators and data collection tools and techniques highlights that they can vary significantly depending on the specific improvement objectives. As emerged from past experiences, performance measurement indicators used to assess the reduction of non-value-added time are the most adopted in the target setting. These include Lead Time, Waiting Time, and Cycle Time. In terms of the number of papers, two productivity indicators follow: the number of patients under care and the Value-Added Ratio. However, a significant distinction lies in the usage and variability of indicators between the timeliness and efficiency categories. While the timeliness category has a few widely used indicators, the efficiency category exhibits a higher variability of indicators. This highlights the complexity of identifying the appropriate indicator, depending on the context. As previously reported, the literature provides limited evidence on performance measurement indicators for effectiveness, patient-centeredness, employee work-life balance, and safety. Regarding effectiveness, indicators have been identified to measure new diagnoses and preventive activities. This highlights the potential impact of HLM in LCOH settings, supporting proximity care, facilitating early diagnosis, and enhancing prevention. These aspects are particularly important when considering the LTC pathways of the aging population and chronic patients [1, 5, 6].

This study emphasizes that data collection methods must be carefully selected, taking into account the improvement objectives and the specific characteristics of performance measurement indicators. To address multiple purposes, these methods can be integrated and adapted to suit specific requirements, enabling the assessment of diverse and comprehensive care quality improvement objectives. Moreover, it emerges that in HLM improvement projects within TAH, data collection for performance measurement lacks standardization and reliable data retrieval approaches, as data are usually gathered through field observations (Fig. 5). The limited evidence regarding long-term monitoring initiatives indicates that the adoption of tools and techniques for the automated and digitalized measurement of indicators remains scarce. This study suggests that field observations are often relied upon, possibly due to the lack or inadequacy of robust digital information systems, Electronic Health Records, and quality registries within LCOH to support performance measurement activities. Without a structured digital system to collect and store data, organizations face significant challenges in defining objectives, identifying indicators, and tracking performance over time due to the limited access to data and effective tools for reporting and dashboard creation [92, 93]. However, continuous improvement can only be achieved through consistent performance monitoring over time, guaranteeing the long-term sustainability of the outcomes [31].

This study has also some limitations that should be considered. Firstly, the exclusion of grey literature, while aiming to strengthen methodological rigor and ensure high-quality standards, may have limited the comprehensiveness of the review. Secondly, the dynamic evolution of the field may have resulted in the omission of recent contributions that are not yet accessible through academic databases. Third, the identified literature is predominantly concentrated in the United States, with limited evidence regarding the implementation of HLM in LCOH settings in other regions, particularly within European public healthcare systems. In future, broadening the scope of research in other areas would be especially valuable, given the economic, managerial, and organizational constraints typical of public healthcare facilities.

## Conclusion

The current literature review provides a structured synthesis of care quality improvement objectives, performance measurement indicators, and data collection tools and techniques adopted in HLM projects within LCOH. It provides valuable insights into the performance measurement indicators that should be adopted in accordance with the specific improvement objectives of HLM projects, while also highlighting the most commonly used data collection tools and techniques. These findings can support the implementation of HLM improvement initiatives within TAH, contribute to strengthening the territorial healthcare system, and enhance the quality of care. Moreover, they support the knowledge advancement in this field, which is a fundamental care setting for the aging population. Indeed, this setting remains underexplored despite the urgent need to ensure high standards of care quality. Moreover, this research identifies four key areas of limited evidence that require further investigation:

### Limited evidence on addressing the challenge of sustaining improvements over time

The literature analyzed indicates limited evidence on projects that sustain improvements over time. Only a limited number of papers implement control plans to conduct long-term measurements and monitoring. There is a notable call for studies investigating the implementation of long-term control plans, particularly with respect to their design and implementation for improvement measurement.

### Limited evidence of healthcare sustainability being addressed across economic, social, and environmental dimensions

This analysis highlights that sustainability in HLM implementations within LCOH is not yet addressed in a balanced manner across its economic, social, and environmental aspects. The economic dimension is the most extensively addressed, primarily through efforts to enhance efficiency and optimize resource utilization. In contrast, there is limited evidence of improvement objectives supported by performance measurement indicators specifically designed to assess environmental impact. Similarly, although some aspects of social sustainability are addressed, they could be further explored by adopting performance measurement indicators into HLM implementations to assess clinical outcomes and maintain high standards of care.

### Limited evidence in the use of automated and digitalized systems for data collection, storage, and retrieval

This analysis highlights the limited evidence regarding the adoption of automated and digitalized systems for data collection, storage, and retrieval within HLM projects implemented in LCOH settings. In most studies, dashboards or other periodic monitoring techniques are not designed and integrated as part of the control and monitoring activities. Moreover, there is limited evidence of performance measurements that rely on structured and consistent data warehouses, underscoring the scant adoption of performance measurement systems in the context analyzed.

### Limited evidence addressing all dimensions of care quality

The current literature review highlights very few projects in which the implementation of HLM integrated improvement objectives related to enhancing patients’ safety. None of the reviewed studies describe a comprehensive and structured system for monitoring clinical risks. Furthermore, only a few papers address patients’ and employees’ satisfaction, with measurements typically limited to one-time surveys that are rarely repeated over time. No paper sets care integration and equity objectives, underscoring the lack of attention to these critical issues. In fact, given the territorial nature of TAH settings, collaboration with other services is essential to ensure universal access to care.

### Limited evidence on HLM improvement projects in TAH

The current scoping literature review on the implementation of HLM projects in TAH settings has highlighted a limited number of studies on this topic, considering both theoretical and empirical research.

In conclusion, the literature review provides an in-depth analysis of HLM implementations within a specific context, addressing the need highlighted in previous studies for more target and context-specific research [94]. The identified gaps are related to the TAH setting but reflect limitations in HLM initiatives observed in other healthcare contexts. From a sustainability perspective, previous studies across various settings have highlighted the widespread challenges of sustaining improvements over time [95] and have outlined significant gaps in the adoption of performance measurement systems within multiple healthcare organizations worldwide [35]. Moreover, a systemic approach to quality is frequently emphasized as a critical factor in ensuring universal healthcare coverage, especially within territorial care settings. This perspective would promote a strong motivation toward HLM initiatives that address quality objectives, encompassing not only timeliness and efficiency but also integration, patient-centeredness, safety, equity, and effectiveness. However, this broader approach to quality is not widely adopted in HLM improvement projects across different healthcare settings [16, 94, 96].

This research highlights the need to expand knowledge of HLM implementation in TAH settings through theoretical and empirical studies. It further highlights the importance of exploring quality improvement from multiple perspectives, with a particular focus on patient safety and clinical risk. Lastly, it stresses the need for future research to focus on the adoption of performance measurement systems in this context, with the final aim of supporting HLM projects and the long-term sustainability of the improvement achieved.

## Electronic supplementary material

Below is the link to the electronic supplementary material.

Supplementary material 1

## Data Availability

The data used for the Scoping Literature Review was retrieved from scientific databases as specified in the manuscript.
