# Supplementary material for: Sustainable care quality improvement: a scoping literature review of performance measurement in lean healthcare implementations
Source: BMC Health Serv Res. 2025 Nov 7;25:1452. doi: 10.1186/s12913-025-13598-5 (PMC12595819; doi:10.1186/s12913-025-13598-5)
Supplement: Supplementary file 1 — Supplementary material 1 [file 12913_2025_13598_MOESM1_ESM.docx]

Additional file 1: Final literature review database. Table by authors.

| **Authors** | **Year** | **Title** | **Journal** | **Study Methodology** |
| --- | --- | --- | --- | --- |
| Aissaoui et al. | 2022 | A BPMN-VSM based process analysis to improve the efficiency of multidisciplinary outpatient clinics | Production Planning and Control | Action Research |
| Al Hroub et al. | 2019 | Improving the Workflow Efficiency of an Outpatient Pain Clinic at a Specialized Oncology Center by Implementing Lean Principles | Asia-Pacific Journal of Oncology Nursing | Case Study |
| Al-Zain et al. | 2019 | Implementing Lean Six Sigma in a Kuwaiti private hospital | International Journal of Health Care Quality Assurance | Case Study |
| Aleem | 2013 | Translating 10 lessons from lean six sigma project in paper-based training site to electronic health record-based primary care practice: Challenges and opportunities | Quality Management in Healthcare | Case Study |
| Aleem et al. | 2015 | Depression screening optimization in an academic rural setting | International Journal of Health Care Quality Assurance | Case Study |
| Astiena et al. | 2022 | Digitalization of Outpatient Services Based on Lean Management to Reduce Waiting Time in Government Hospital | International Journal on Advanced Science Engineering Information Technology | Case Study |
| Bakhai et al. | 2024 | Strategies to Improve COVID-19 Vaccination in a Pregnant, Marginalized Population: Quality Improvement Project | American Journal of Public Health | Case Study |
| Bakhai et al. | 2024 | Lean Six Sigma quality improvement approach to implement clinical practice guidelines for prescribing opiates for chronic pain in a primary care setting | BMJ Open Quality | Case Study |
| Baril et al. | 2016 | Use of a discrete-event simulation in a Kaizen event: A case study in healthcare | European Journal of Operational Research | Case Study |
| Bhat & Jnanesh | 2014 | Application of Lean Six Sigma methodology to reduce the cycle time of outpatient department service in a rural hospital | International Journal of Healthcare Technology and Management | Case Study |
| Cheung et al. | 2016 | No More Waits and Delays: Streamlining Workflow to Decrease Patient Time of Stay for Image-guided Musculoskeletal Procedures | Radiographics | Case Study |
| Ciulla et al. | 2018 | Lean six sigma techniques to improve ophthalmology clinic efficiency | Retina | Case Study |
| Damle et al. | 2016 | Elimination of waste: creation of a successful Lean colonoscopy program at an academic medical center | Surgical Endoscopy and Other Interventional Techniques | Case Study |
| Davies et al. | 2019 | Optimizing nursing time in a day care unit: Quality improvement using Lean Six Sigma methodology | International Journal of Quality in Healthcare | Case Study |
| Demirli et al. | 2021 | Using lean techniques and discrete-event simulation for performance improvement in an outpatient clinic | International Journal of Lean Six Sigma | Case Study |
| Duska et al. | 2015 | Lean methodology improves efficiency in outpatient academic Gynecologic Oncology clinics | Gynecologic Oncology | Case Study |
| Fields et al. | 2018 | Using Lean methodologies to streamline processing of requests for durable medical equipment and supplies for children with complex conditions | Healthcare | Case Study |
| Gerard et al. | 2021 | Applying Lean Six Sigma to Improve Depression Screening and Follow-Up in Oncology Clinics | Journal for healthcare quality: official publication of the National Association for Healthcare Quality | Case Study |
| Gijo & Antony | 2014 | Reducing patient waiting time in outpatient department using lean six sigma methodology | Quality and Reliability Engineering International | Case Study |
| Hung et al. | 2019 | Sustainment of lean redesigns for primary care teams | Quality Management in Healthcare | Survey |
| Hung et al. | 2019 | Contextual Conditions and Performance Improvement in Primary Care | Quality Management in Healthcare | Survey |
| Igoe et al. | 2024 | Implementing Person-Centred Lean Six Sigma to Transform Dermatology Waiting Lists: A Case Study from a Major Teaching Hospital in Dublin, Ireland | Sci | Case Study |
| Kam et al. | 2021 | Using Lean Six Sigma techniques to improve efficiency in outpatient ophthalmology clinics | BMC Health Services Research | Case Study |
| Kollipara et al. | 2021 | Improving Diabetic Retinopathy Screening Among Patients With Diabetes Mellitus Using the Define, Measure, Analyze, Improve, and Control Process Improvement Methodology | Journal for healthcare quality: official publication of the National Association for Healthcare Quality | Case Study |
| Kovach & Flores | 2021 | Streamlining admissions to outpatient substance use treatment using lean methods | Journal of Substance Use | Case Study |
| Kovach & Ingle | 2019 | Using Lean Six Sigma to Reduce Patient Cycle Time in a Nonprofit Community Clinic | Quality Management in Healthcare | Case Study |
| Laganga | 2011 | Lean service operations: Reflections and new directions for capacity expansion in outpatient clinics | Journal of Operations Management | Action Research |
| Lobo-Prat et al. | 2025 | Designing an integrated care pathway for spondyloarthritis: A Lean Thinking approach | Journal of Evaluation in Clinical Practice | Case Study |
| Maitre et al. | 2020 | Network implementation of guideline for early detection decreases age at cerebral palsy diagnosis | Pediatrics | Case Study |
| McDermott et al. | 2013 | Restructuring of the Diabetes Day Centre: A pilot lean project in a tertiary referral centre in the West of Ireland | BMJ Quality and Safety | Case Study |
| Morell-Santandreu et al. | 2021 | A model for the implementation of lean improvements in healthcare environments as applied in a primary care center | International Journal of Environmental Research and Public Health | Action Research |
| Muharam & Firman | 2022 | Lean Management Improves the Process Efficiency of Controlled Ovarian Stimulation Monitoring in IVF Treatment | Journal of Healthcare Engineering | Action Research |
| Naidoo & Mahomed | 2021 | Impact of Lean on patient cycle and waiting times at a rural district hospital in KwaZulu-Natal | African Journal of Primary Health Care and Family Medicine | Action Research |
| Paccagnella et al. | 2012 | Quality improvement for integrated management of patients with type 2 diabetes (PRIHTA project stage 1) | Quality Management in Healthcare | Case Study |
| Salam & Khan | 2016 | Value creation through lean management: A case study of healthcare service operations | International Journal of Services and Operations Management | Case Study |
| Schoonhoven et al. | 2011 | Quality quandaries: Streamlining the path to optimal care for cardiovascular patients | Quality Engineering | Case Study |
| Shaban et al. | 2022 | Integration of simulation modelling and lean management to improve patient flow at outpatient clinics | International Journal of Productivity and Quality Management | Case Study |
| Skeldon et al. | 2014 | Lean methodology improves efficiency in outpatient academic uro-oncology clinics | Urology | Case Study |
| Smith et al. | 2011 | Thinking lean: implementing DMAIC methods to improve efficiency within a cystic fibrosis clinic. | Journal for healthcare quality: official publication of the National Association for Healthcare Quality | Case Study |
| Sullivan et al. | 2022 | Redesigning patient flow in orthopedics and radiology clinics via a three-phase 'Kaizen' improvement approach and interrupted time series analysis | International Journal for Quality in Health Care | Case Study |
| Swee et al. | 2020 | Development and implementation of a Telenephrology dashboard for active surveillance of kidney disease: A quality improvement project | BMC Nephrology | Case Study |
| Szekeres et al. | 2025 | Improving access to outpatient computed tomography | Current Problems in Diagnostic Radiology | Case Study |
| Van Leijen-Zeelenberg et al. | 2016 | Using Lean Thinking at an otorhinolaryngology outpatient clinic to improve quality of care | Laryngoscope | Survey |
| van Lent et al. | 2009 | Improving the efficiency of a chemotherapy day unit: Applying a business approach to oncology | European aJournal of Cancer | Case Study |
| Vidal-Carreras et al. | 2022 | Applying Value Stream Mapping to Improve the Delivery of Patient Care in the Oncology Day Hospital | International Journal of Environmental Research and Public Health | Case Study |
| White et al. | 2021 | Use of Lean Methodologies in Outpatient Urology Clinic | Urology Practice | Case Study |
| Wilson et al. | 2020 | Using Lean Six Sigma to Improve Pneumococcal Vaccination Rates in a Veterans Affairs Rheumatology Clinic | Journal for healthcare quality: official publication of the National Association for Healthcare Quality | Case Study |
